# The Association Between Peak IL-6 Levels, In-Hospital Mortality, and Selected Risk Factors in Patients Hospitalized for COVID-19 at the Temporary Hospital in Gdansk—A Retrospective Cohort Study

**DOI:** 10.3390/jcm15155896

**Published:** 2026-07-28

**Authors:** Dariusz Kostrzewa, Anna Justyna Milewska, Aleksandra Dorobek, Michal Marczak, Kacper Wróbell, Piotr Wroblewski, Krystyna Paszko, Remigiusz Kozlowski

**Affiliations:** 1 Department of Management and Logistics in Healthcare, Medical University of Lodz, 90-419 Lodz, Poland; remigiusz.kozlowski@umed.lodz.pl; 2 Copernicus Podmiot Leczniczy Ltd., 80-803 Gdansk, Poland; adorobek@copernicus.gda.pl (A.D.); p.wroblewski@copernicus.gda.pl (P.W.); kpaszko@copernicus.gda.pl (K.P.); 3 Department of Biostatistics and Medical Informatics, Medical University of Bialystok, 15-295 Bialystok, Poland; anna.milewska@umb.edu.pl; 4 Department of Innovation, Merito University in Poznan, 61-895 Poznan, Poland; michal.marczak@warszawa.merito.pl; 5 District Sanitary and Epidemiological Station in Bialystok, 15-099 Bialystok, Poland; kacperwrobel@gmail.com

**Keywords:** COVID-19, interleukin-6, in-hospital mortality, temporary hospital, retrospective cohort study

## Abstract

**Introduction:** Interleukin-6 is one of the key mediators of the inflammatory response and has repeatedly been associated with severe COVID-19 and an increased risk of death. However, its clinical significance depends on the timing of measurement, the characteristics of the study population, and the organizational context of care. Data on the significance of IL-6 in temporary hospital settings remain limited. **Objective:** The objective of this study was to assess the association between the highest available interleukin-6 (IL-6) concentration recorded during hospitalization and in-hospital mortality among patients hospitalized for COVID-19 at the Temporary Hospital in Gdansk. Additional objectives were to assess the ability of IL-6 to discriminate in-hospital mortality, determine an exploratory cutoff point for the study cohort, and assess selected clinical factors associated with elevated IL-6 concentrations. **Materials and Methods:** A retrospective, single-center cohort study was conducted involving 1214 patients hospitalized for COVID-19 during two periods of operation of the Temporary Hospital in Gdansk. IL-6 measurements were performed selectively, at the discretion of the attending physician; therefore, IL-6 analyses were conducted in a subcohort of 171 patients with an available result. The highest available IL-6 concentration recorded during each patient’s hospitalization was used in the analysis. Patients with and without an IL-6 measurement were compared. The association between IL-6 and in-hospital mortality was assessed using ROC analysis and a multivariable logistic regression model in which IL-6 was analyzed as a continuous variable after log_2_ transformation. **Results:** A total of 230 in-hospital deaths were recorded in the entire cohort. IL-6 was measured in 171 patients. Patients with an IL-6 measurement differed from the remaining hospitalized patients in terms of age, sex, year of hospitalization, mortality, length of hospital stay, and the prevalence of respiratory disease, indicating the clinically selective nature of this subcohort. ROC analysis for IL-6 levels yielded an AUC of 0.73, and the cutoff point determined using the Youden method was 94.3 pg/mL. In the multivariable logistic regression model, log_2_-transformed IL-6 remained associated with in-hospital mortality after adjustment for age, year of hospitalization, and respiratory disease. A two-fold increase in IL-6 concentration was associated with an approximately 27.5% increase in the odds of death. **Conclusions:** The highest available IL-6 concentration recorded during hospitalization was associated with in-hospital mortality in the analyzed subcohort of patients hospitalized for COVID-19. The findings support the relevance of IL-6 as a marker of an intensified inflammatory response during hospitalization, but should not be interpreted as validation of IL-6 as a standalone prognostic tool measured at hospital admission.

## 1. Introduction

COVID-19 was characterized by substantial variability in its clinical course, ranging from paucisymptomatic infection to severe respiratory failure, multi-organ failure, and death. Since the beginning of the pandemic, markers of the inflammatory response have attracted particular interest because they could reflect the intensity of the disease process and support assessment of the risk of an unfavorable hospital course. Early in the pandemic, clinicians observed an association between elevated interleukin-6 (IL-6) concentrations and an unfavorable course of COVID-19. These clinical observations were reflected in studies assessing the relationship between the concentration of this marker and disease severity, mortality, and clinical and laboratory parameters evaluated at hospital admission and during subsequent treatment [1,2].

Interleukin-6 is one of the key mediators of the inflammatory response and plays an important role in activation of the acute-phase response. It is a pleiotropic cytokine involved in the regulation of inflammatory, immune, and metabolic responses. It is produced by numerous cell types, including monocytes, macrophages, T lymphocytes, fibroblasts, endothelial cells, epithelial cells, and adipocytes, particularly in response to infection, tissue damage, or activation of the immune system [3]. One of its key actions is stimulation of hepatocytes to synthesize acute-phase proteins, including C-reactive protein, fibrinogen, and hepcidin, which gives it an important role in the systemic inflammatory response [4]. During COVID-19, increased IL-6 concentrations have repeatedly been associated with a more severe disease course, a greater risk of respiratory failure, poorer prognosis, and a higher risk of death. The significance of this parameter also depends on the clinical context, timing of measurement, frequency of testing, and characteristics of the study population. As a mediator of an intensified inflammatory response, IL-6 is substantially associated with the phenomenon referred to as cytokine storm [5,6].

Meta-analyses indicate that, despite the significant value of IL-6 as a marker of disease severity in patients with COVID-19, its interpretation should take into account the patient’s clinical condition, the timing of measurement, and the results of other inflammatory markers because its standalone prognostic value for mortality remains dependent on the study context [6,7]. Similar observations were also reported in an analysis of inflammatory markers in patients hospitalized for COVID-19, in which IL-6 differentiated patients with more severe and milder disease courses and remained associated with the risk of death [8]. A systematic review showed that IL-6 concentrations were approximately 2.9 times higher in patients with complicated COVID-19 than in those with an uncomplicated course, further supporting its significance as a marker of an unfavorable disease course [9]. Of particular importance is the distinction between measurements performed at admission and maximum values recorded during hospitalization, which may better reflect the peak intensity of the inflammatory response but are also more susceptible to the influence of treatment and monitoring intensity. Increased IL-6 concentrations were also observed together with increased concentrations of CRP, ferritin, D-dimer, LDH, and procalcitonin [10,11,12,13].

The risk of severe COVID-19 is also influenced by patient characteristics present before the illness, including age, sex, and comorbidities. The analyzed material included cancer, diabetes, cardiovascular disease, and respiratory disease. These factors are associated not only with an increased risk of death but also with the intensity of the inflammatory response, one marker of which is an elevated IL-6 concentration [14,15,16]. Therefore, interpreting IL-6 measurement results without considering the patients’ basic clinical characteristics would be an oversimplification.

Temporary hospitals were an important element of the organizational response of the healthcare system to the rapid increase in hospitalizations during the COVID-19 pandemic. Their purpose was to increase bed availability for patients infected with SARS-CoV-2, particularly those requiring oxygen therapy, monitoring, and internal medicine treatment, while relieving conventional hospitals [17,18]. The operating conditions of such facilities differed from standard hospital wards, including in terms of admission dynamics, resource availability, eligibility criteria, organization of care, and the profile of patients referred for hospitalization during periods of the greatest epidemic burden. These facilities became not only an organizational solution but also a setting for clinical analyses of the disease course [17]. In addition to the clinical aspect, working conditions of medical staff and the influence of workplace architecture on team functioning were also described [19].

Although the association between IL-6 and unfavorable outcomes in COVID-19 had been analyzed previously, data concerning patients hospitalized in temporary hospitals remain limited. The primary aim of this study was to assess the association between the highest available IL-6 concentration recorded during hospitalization and in-hospital mortality among patients hospitalized for COVID-19 at the Temporary Hospital in Gdansk. Additional aims were to assess the ability of IL-6 to discriminate in-hospital mortality, determine an exploratory cutoff point for the study cohort, and assess selected clinical factors associated with elevated IL-6 concentrations.

## 2. Materials and Methods

### 2.1. Study Design

This was a retrospective, single-center cohort study based on an analysis of the medical records of patients hospitalized for COVID-19 at the Temporary Hospital in Gdansk. The analysis covered two periods of the facility’s operation: from 8 March 2021 to 30 June 2021, and from 19 January 2022 to 31 March 2022. The study was prepared in accordance with the STROBE recommendations for cohort studies.

### 2.2. Setting

The study was based on data from the medical records of patients hospitalized at the Temporary Hospital in Gdansk. The hospital functioned as a facility intended for the hospitalization of patients with COVID-19. The study material comprised the complete medical records of 1214 hospitalized patients.

The Temporary Hospital, organized in an exhibition and trade fair hall, was prepared for a maximum of 400 patients, including 20 patients requiring mechanical ventilation. Five internal medicine and infectious disease wards were established, each with 96 beds, as well as one 20-bed intensive care unit. The internal medicine wards were equipped with basic patient-monitoring devices and an oxygen system enabling respiratory support. The intensive care unit was equipped with ventilators and equipment enabling treatment of patients in a medically induced coma. For functional and formal-legal reasons, the medical area was divided into 28-bed modules, each of which was supervised around the clock by one physician, two nurses, one paramedic, and four caregivers. Two 10-bed modules were designated in the intensive care unit, each staffed by two physicians (including at least one anesthesiologist), three nurses, two paramedics, and four caregivers.

Patients were admitted to the Temporary Hospital directly from their place of residence or from other medical facilities after being diagnosed with SARS-CoV-2 infection, provided that treatment outside the hospital was not possible. Patients requiring urgent surgical interventions were disqualified from admission. They were referred to surgical isolation and infectious disease wards designated by the provincial governor. The hospital also did not treat patients requiring gynecological and obstetric procedures, particularly pregnant women.

By decision of the Pomeranian provincial governor, surgical hospital wards were designated in which isolation conditions were provided for patients undergoing surgical intervention. These wards provided surgical treatment of acute conditions in general surgery, neurosurgery, urology, gynecology and obstetrics, and orthopedic and trauma surgery.

The Temporary Hospital described in this study served as a key healthcare facility during the 2021/2022 pandemic in the Pomeranian Province. It should be noted that its bed capacity was never fully used, and the maximum number of patients hospitalized simultaneously was approximately 200. At the peak of the facility’s workload, it employed a total of 24 physicians, 43 nurses, 48 paramedics, and 42 caregivers.

### 2.3. Participants

The study included 1214 patients hospitalized for COVID-19 during the analyzed periods (679 patients were hospitalized in 2021 and 535 in 2022). Patients were transported by emergency medical teams, transferred from other hospitals, or presented independently. The primary diagnosis in all patients was SARS-CoV-2 infection.

### 2.4. Endpoints

The primary endpoint of the study was in-hospital death. During the analyzed period, 230 in-hospital deaths were recorded. In-hospital death was defined as the death of a patient that occurred during the hospital stay, that is, during inpatient treatment. This means that the patient died within the medical facility before being discharged home or to another place of care. Acute respiratory failure (J96.0 according to the ICD-10 classification) was most frequently coded as the immediate cause of death. COVID-19, with the virus identified (code U07.1), was reported as a comorbid condition or as the primary cause in every patient.

Additional analyses assessed the occurrence of elevated IL-6 concentrations, defined on the basis of the cutoff point of 94.3 pg/mL determined using the Youden method in ROC analysis. IL-6 measurements were not performed routinely in all patients but selectively, at the discretion of the attending physician. Therefore, all analyses concerning IL-6 should be interpreted as an analysis of the subcohort of 171 patients with an available IL-6 result, rather than as an assessment of a marker used systematically throughout the cohort. The analyzed exposure was defined as the highest available IL-6 concentration recorded in a given patient during hospitalization. In patients with only one available IL-6 result, this value was treated as the highest available concentration.

A total of 213 IL-6 measurements were performed in the subcohort of 171 patients with an available IL-6 result. The median number of IL-6 measurements per patient was 1 (IQR 1–1; range 1–4). One IL-6 measurement was available in 140 patients (81.9%), whereas more than one measurement was performed in 31 patients (18.1%). Among patients with more than one measurement, the median number of measurements was 2 (IQR 2–3; range 2–4). Therefore, the analyzed exposure was defined as the highest available IL-6 concentration recorded during hospitalization, without treating this value as a confirmed biological peak in IL-6 concentration.

### 2.5. IL-6 Measurement

Interleukin-6 concentrations were measured in serum or plasma via an electrochemiluminescence immunoassay (ECLIA), using a quantitative sandwich immunoassay manufactured by Roche Diagnostics (Penzberg, Germany) on a Cobas e 801 analyzer. The measurement was performed as part of routine laboratory diagnostics, in accordance with laboratory procedures and the manufacturer’s instructions. Results were reported in pg/mL.

### 2.6. Covariates

The study included the following patient characteristics present before hospitalization: age, sex, cancer, diabetes, cardiovascular disease, and respiratory disease. The year of hospitalization was analyzed as a variable reflecting differences between epidemic periods. Length of hospital stay was included in descriptive analyses and is not interpreted as a classic outcome variable because it partly represents a consequence of the disease course.

### 2.7. Data Sources and Potential Sources of Bias

The data were derived from routinely maintained medical records. The most important potential source of distortion of the results was the selective nature of IL-6 measurements. This parameter was ordered for clinical reasons, probably more frequently in patients with clinical deterioration, which creates a risk of selection bias and distortion resulting from non-random testing. The analysis did not include disease severity indicators, respiratory parameters, treatment, or a complete panel of additional laboratory test results. The scope of the available data limited the possibility of assessing the independent prognostic value of IL-6 measured at a standardized time point.

### 2.8. Study Size

The study included all patients hospitalized during the analyzed periods; therefore, no formal sample size calculation was performed before the analysis. Analyses concerning IL-6 were conducted in the subcohort of 171 patients with an available measurement of this parameter.

When essential data were missing or incomplete, the patient record was omitted from the relevant analyses. Of the total population of 1244 patients, data loss before the analysis concerned 30 individuals, representing 2.4% of the analyzed population. In each case, these were individuals whose identity had not been established or whose personal data had not been recorded in the system. For rehospitalized patients (admitted to the temporary hospital more than once), data from the most recent stay were included in the analyses. For some foreign nationals, it was not possible to retrospectively reconstruct sex, age, or medical history from the period preceding the stay in the temporary hospital.

### 2.9. Statistical Analysis

The collected data were presented as descriptive statistics: for qualitative data, as the number of observations and percentage, and for quantitative variables, as the median, lower quartile, and upper quartile. Normality of the distribution of quantitative variables was assessed using the Shapiro–Wilk test. Because the analyzed variables were not normally distributed, the Mann–Whitney U test was used for comparisons, and Pearson’s chi-square test or Fisher’s exact test, depending on cell counts, was used for categorized variables. Patients with an IL-6 measurement were compared with patients without an IL-6 measurement to assess potential selection bias. The discriminatory properties of the highest available IL-6 concentration for in-hospital mortality were assessed using ROC analysis, and the cutoff point was determined using the Youden method. Because of the limitations of dichotomizing a continuous variable, logistic regression models assessing the odds of in-hospital death were also performed, in which IL-6 was analyzed as a continuous variable after log_2_ transformation. Age, sex, history of cancer, diabetes, cardiovascular disease, respiratory disease, year of hospitalization, and log_2_ IL-6 were considered in the analysis. The multivariable model included variables associated with death in univariable models: age, log_2_ IL-6, year of hospitalization, and respiratory disease. Analyses concerning IL-6 ≥ 94.3 pg/mL were treated as additional and descriptive. Model goodness of fit was assessed using the Hosmer–Lemeshow test and a calibration curve, and discriminatory ability was assessed on the basis of the area under the ROC curve. A significance level of 0.05 was adopted. Analyses were performed using Stata/SE 18.0 (StataCorp LLC, College Station, TX, USA).

### 2.10. Ethical Considerations

The study was approved by the Bioethics Committee of the Regional Medical Chamber in Gdansk, approval number KB-36/22, dated 12 July 2022. The approval concerned the study conducted by the principal investigator, Dariusz Kostrzewa, MD, at Copernicus Sp. z o.o. in Gdansk. The Committee raised no objections to conducting the study in accordance with the submitted documents. The exemption from the requirement to obtain informed consent from patients resulted from the retrospective nature of the analysis.

## 3. Results

### 3.1. Characteristics of the Analyzed Cohort

The study included 1214 patients hospitalized for COVID-19. A total of 230 in-hospital deaths were recorded in the entire cohort, representing 18.95% women accounted for 45.39% of the participants, and men accounted for 54.61%. The median age was 69.0 years, and the median length of hospital stay was 10.0 days. Cardiovascular disease was present in 69.19% of patients, diabetes in 24.79%, respiratory disease in 25.29%, and cancer in 7.74%. Information about the study group is summarized in Table 1.

### 3.2. Frequency of IL-6 Measurement

IL-6 was measured in 171 patients. Measurements were performed more frequently in 2021 than in 2022 (*p* < 0.0001). In 2021, IL-6 was measured in 22.77% of patients, and in 2022 in 5.61%. An association was observed between the frequency of IL-6 measurement and death (*p* = 0.0042). Among patients who died, 20% had an IL-6 measurement, whereas among those who survived, IL-6 was measured in 12.7%. Viewed from the opposite direction, 46 of 171 patients with an IL-6 measurement died (26.9%), whereas 184 of 1043 patients without an IL-6 measurement died (17.6%). The distribution of deaths among patients with and without IL-6 measurements is presented in Table 2.

There was also a significant association between the frequency of IL-6 measurement and sex (*p* < 0.0001). IL-6 was measured in 9.62% of women and 17.8% of men. Patients in whom IL-6 was measured were, on average, 64 years old, whereas those in whom it was not measured were older and were, on average, 70 years old. The age difference was statistically significant (*p* < 0.0001). Among patients with respiratory disease, IL-6 was measured in 26.71%, whereas among those without respiratory disease, it was measured in 9.81%; this association was statistically significant (*p* < 0.0001). There was no statistically significant association between IL-6 measurement and diabetes, cardiovascular disease, or a history of cancer. IL-6 measurement was associated with the length of treatment (*p* < 0.0001). Patients with an IL-6 measurement were treated for an average of 14 days, whereas those without an IL-6 measurement were treated for 10 days. These results indicate that the decision to perform the test was not random. The IL-6 subcohort should therefore be regarded as clinically selected rather than as a representative sample of all hospitalized patients. Data on comorbidity are summarized in Table 3.

**Figure 1:**
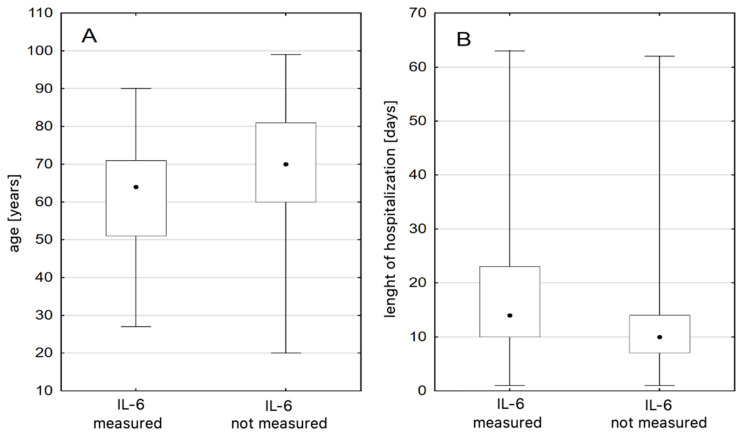
Distribution of age [years] (**A**) and length of hospital stay [days] (**B**) in patients with and without an IL-6 measurement. The dots represent the median values of the parameters shown. The boxes encompass the range between the first and third quartiles. The lines illustrate the range between the smallest and biggest values.

The age distribution and length of hospital stay for the analyzed group of patients are shown in Figure 1. 

### 3.3. Discriminatory Properties of IL-6 for In-Hospital Mortality

In the subcohort of patients with an IL-6 measurement, 46 in-hospital deaths were recorded, representing 26.9%. Women accounted for 31.0% of the participants and men for 69.0%. The median age was 64.0 years, and the median length of hospital stay was 14.0 days. Cardiovascular disease was present in 70.8% of patients, diabetes in 28.7%, respiratory disease in 48.0%, and cancer in 5.3%.

In the ROC analysis assessing the ability of the highest available IL-6 concentration to discriminate between patients who died and those who survived, an AUC of 0.73 was obtained, with a 95% confidence interval from 0.651 to 0.814. Based on the Youden index, a cutoff point of 94.3 pg/mL was determined. This threshold was treated as an exploratory value specific to the analyzed cohort rather than as a validated clinical decision point.

**Figure 2:**
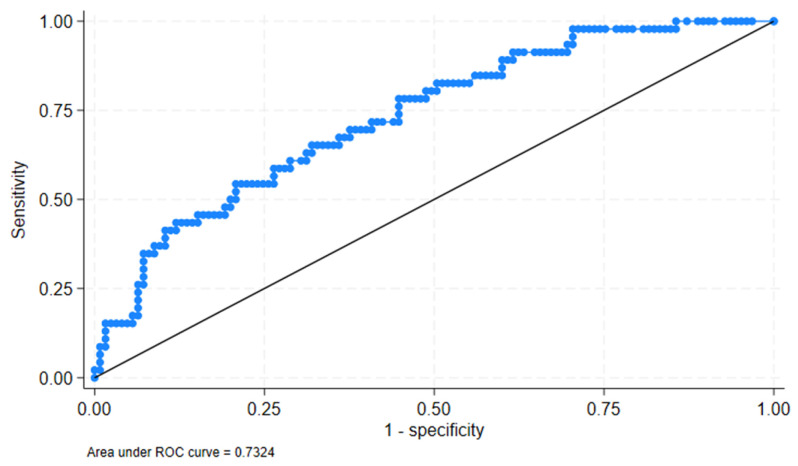
ROC curve and determination of the IL-6 cutoff point above which its association with death was confirmed.

Analysis of the relationship between high and low IL-6 levels showed a statistically significant association with in-hospital mortality (*p* < 0.0001). Among patients who died, high IL-6 was found in 54.3%, whereas among patients who survived, it was found in 20.8%. Viewed from the opposite direction, 49% of patients with high IL-6 died, whereas 18% of patients with lower IL-6 died. In univariable analysis, IL-6 ≥ 94.3 pg/mL was associated with higher odds of in-hospital death (OR 4.53; 95% CI 2.20–9.34; *p* < 0.001). Odds ratios were also used to analyze the association between two levels of IL-6 and death. Figure 2 shows a ROC curve, and Table 4 presents the numerical data corresponding to the distribution of the population according to the calculated cutoff point.

### 3.4. Association of High IL-6 with Selected Patient Characteristics

Comparison of patients with high and low IL-6 readings showed a statistically significant association between IL-6 level and the presence of respiratory disease (*p* < 0.0001). Among patients with respiratory disease, 48% had high IL-6, whereas among those without respiratory disease, 13% had high IL-6. A statistically significant difference was also observed between the ages of patients with high and low IL-6 (*p* = 0.006). Patients with low IL-6 were, on average, 61 years old, whereas those with high IL-6 were 68 years old.

**Figure 3:**
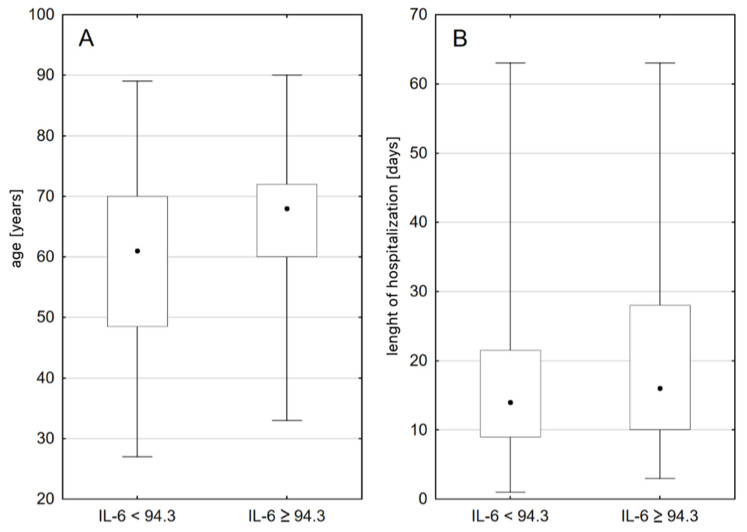
Distribution of age [years] (**A**) and length of hospital stay [days] (**B**) in the IL-6 < 94.3 pg/mL and IL-6 ≥ 94.3 pg/mL groups. The dots represent the median values of the parameters shown. The boxes encompass the range between the first and third quartiles. The lines illustrate the range between the smallest and biggest values.

No significant association was found between IL-6 level and year of hospitalization, sex, history of cancer, diagnosed diabetes, or cardiovascular pathology. No significant difference was also found in the length of treatment between patients with high and low IL-6 levels. The complete numerical data obtained in this analysis are presented in Table 5. Figure 3 provides a graphical representation of the age distribution of patients and the length of their hospital stays.

### 3.5. Model of the Odds of Death

Logistic regression models assessing the odds of in-hospital death were estimated in the subcohort of patients with a measured IL-6 concentration. The analysis included variables available at admission to the ward: age, sex, history of cancer, diabetes, cardiovascular disease, respiratory disease, year of hospitalization, and IL-6 concentration after log_2_ transformation. The log_2_ transformation allows interpretation of the odds ratio for a two-fold increase in IL-6 concentration.

In univariable models, age, log_2_ IL-6, year of hospitalization, and respiratory disease were associated with in-hospital death. These variables were subsequently included in the multivariable model.

In the multivariable model, older age, higher log_2_ IL-6 concentration, year of hospitalization 2021, and coexisting respiratory disease were associated with increased odds of in-hospital death. A two-fold increase in IL-6 concentration was associated with an approximately 27.5% increase in the odds of death (OR 1.275; 95% CI 1.075–1.512; *p* = 0.005). The analytical data for both variants are summarized in Table 6.

Based on the multivariable model, respiratory disease was associated with approximately 7.9-fold higher odds of in-hospital death. Each additional year of age was associated with an approximately 5.8% increase in the odds of death. The odds of death in 2021 were higher than in 2022.

**Figure 4:**
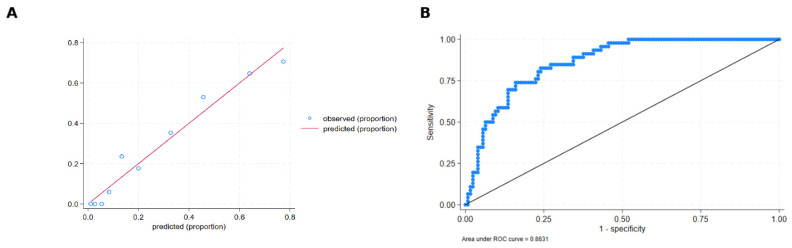
Diagnostics of the model of the odds of death: calibration curve (**A**) and ROC curve (**B**).

Model diagnostics did not indicate significant lack of fit. The Hosmer–Lemeshow test yielded χ^2^ = 4.22; *p* = 0.9368. The area under the ROC curve for the model’s linear predictor was 0.8631 (95% CI 0.8078–0.9184). These results should be interpreted as an assessment of model fit and discriminatory ability in the analyzed subcohort, rather than as validation of a predictive tool for clinical use. Figure 4 provides a graphical representation of the model described above.

## 4. Discussion

The study showed that the highest available interleukin-6 concentration recorded during hospitalization was associated with in-hospital mortality in the subcohort of patients with an available measurement of this parameter. This relationship was evident in ROC analysis and in the multivariable logistic regression model, in which IL-6 was analyzed as a continuous variable after log_2_ transformation. After adjustment for age, year of hospitalization, and respiratory disease, log_2_ IL-6 remained associated with in-hospital mortality. A two-fold increase in IL-6 concentration was associated with an approximately 27.5% increase in the odds of death. The results are consistent with the available literature indicating that higher IL-6 concentrations accompany a more severe course of COVID-19 and a poorer prognosis. In the meta-analysis by Coomes et al. [9], IL-6 concentrations were significantly higher in patients with complicated COVID-19 than in those with an uncomplicated course. Similarly, later meta-analyses demonstrated an association of higher IL-6 levels with more severe disease, intensive care unit stay, and death (Chang et al. [20]; Jafrin et al. [7]). Our observations are therefore consistent with the dominant direction of previous studies while extending them with data from a population treated in a temporary hospital.

The significance of IL-6 in COVID-19 also has a biological basis. This cytokine plays an important role in activation of the acute-phase response and is associated with the intensity of the systemic inflammatory response observed in patients with severe infection. In this context, high IL-6 concentrations may be regarded as a laboratory marker of an intensified inflammatory response accompanying an unfavorable clinical course (Hojyo et al. [21]). Mobilization of the leukocyte system, expressed by numerical ratios including the neutrophil-to-lymphocyte ratio, lymphocyte-to-monocyte ratio, and platelet-to-lymphocyte ratio, was analyzed in patients at the temporary hospital in Bialystok, demonstrating predominance of neutrophilia in patients with severe infection and a risk of death (Dymicka-Piekarska et al. [22]).

Interpretation of the results requires consideration of the definition of the analyzed exposure. The study used the highest available IL-6 concentration recorded during hospitalization rather than a value measured at a standardized time point, for example at hospital admission. This definition may reflect the maximum observable intensity of the inflammatory response in the clinical documentation but does not correspond to a conventional prognostic assessment performed at the beginning of hospitalization. Therefore, the results should be interpreted as an association between the intensity of the inflammatory response during hospitalization and in-hospital mortality, rather than as confirmation of the independent prognostic value of IL-6 measured at admission.

The association of high IL-6 levels with age and respiratory disease should also be noted. The age-related relationship is consistent with previous observations indicating that older patients more frequently develop severe COVID-19 and are characterized by a more intense inflammatory profile (Chang et al. [20]; Jafrin et al. [7]). The observed association with respiratory disease may reflect the particular susceptibility of this group to a more severe course of infection and a more intense inflammatory response. A more severe course of SARS-CoV-2 infection in patients burdened, among other conditions, by respiratory and cardiovascular disease was analyzed by Kostrzewa et al. [23]. By demonstrating the influence of respiratory pathology on IL-6 levels, an increased mortality among patients burdened by diseases in this organ group should indirectly be expected. Studies by Chourasia et al. [24] concerning cardiac burdens in patients diagnosed with COVID-19 also indicate an arrhythmogenic effect of the virus. Cardiovascular decompensation was one of the main indications for hospitalization in temporary hospitals, shaping the structure of the analyzed cohort. The relationship between activation of the immune system, manifested among other things by increased IL-6 levels, and the overall condition of the cardiovascular system therefore receives further support.

The presented results support interpretation of IL-6 as an element of a broader assessment of patients hospitalized for COVID-19, particularly when monitoring patients with a more severe disease course. However, the cutoff point of 94.3 pg/mL should be interpreted cautiously. It was derived and evaluated in the same sample, which may lead to overestimation of its discriminatory ability. Dichotomization of a continuous variable is also associated with loss of some information. Therefore, the threshold of 94.3 pg/mL remains an exploratory result specific to the analyzed cohort rather than a universal threshold or clinical decision point [25].

Another feature distinguishing this study from a number of publications devoted to the course of COVID-19 infection is the nature of the analyzed population. The study was conducted in a temporary hospital, that is, under organizational conditions that played an important role during successive waves of the pandemic but remain less well described in the literature than conventional hospital settings. The presented findings therefore supplement the available data on the course of COVID-19 in real-world clinical practice in this type of facility (Wolszczak-Biedrzycka et al. [8]).

In summary, the findings indicate that high maximum IL-6 concentrations accompany a more severe course of COVID-19 and in-hospital death. Interleukin-6 should, however, be interpreted not as a standalone decisive marker but as an element of a broader clinical assessment that takes into account the patient’s condition, comorbidities, and the dynamics of the hospital course. Further studies should verify these observations in independent cohorts, taking into account a standardized time point for IL-6 measurement and more complete clinical data (Coomes et al. [9]; Chang et al. [20]; Jafrin et al. [7]). Regardless of the associations described in the literature between other biochemical parameters (CRP, ferritin, D-dimer, LDH, and procalcitonin) and the clinical condition of patients, IL-6 is regarded by many authors as a mediator of significant diagnostic and prognostic value [10,25].

IL-6 should be interpreted in the context of other clinical and laboratory data. In the literature, a more severe course of COVID-19 has also been associated with increased CRP, ferritin, D-dimer, LDH, and procalcitonin concentrations, lymphopenia, and an increased neutrophil-to-lymphocyte ratio [10,25]. However, the present study was not designed as a comparison of the diagnostic or prognostic value of different biomarkers.

## 5. Limitations

IL-6 was measured selectively, based on clinical judgment, rather than systematically in all patients. Comparison of patients with and without an IL-6 measurement showed significant differences between the groups, confirming the clinically selective nature of the IL-6 subcohort. Therefore, findings concerning IL-6 should not be generalized to the entire population of patients hospitalized for COVID-19. The analyzed value was the highest available IL-6 concentration recorded during hospitalization rather than a measurement performed at a uniform time point. An analysis of the number of IL-6 measurements per patient did not provide complete information regarding the exact timing of the peak value in relation to the course of the disease. Therefore, it was not possible to analyze IL-6 kinetics over time or confirm that the highest available value corresponded to the actual biological peak of the inflammatory response. The available dataset did not include detailed respiratory parameters, oxygen requirements, severity scores, BMI, detailed treatment, vaccination status, or the exact timing of IL-6 measurement in relation to the disease course. The cutoff point of 94.3 pg/mL was determined and evaluated in the same cohort and should therefore be regarded as exploratory and specific to the analyzed population. The study was retrospective and single-center and was conducted under the particular organizational conditions of a temporary hospital during the COVID-19 pandemic.

## 6. Conclusions

In this retrospective cohort study involving patients hospitalized for COVID-19 in a temporary hospital, the highest available IL-6 concentration recorded during hospitalization was associated with in-hospital mortality in the subcohort of patients in whom this parameter was measured.

This association persisted when IL-6 was analyzed as a continuous variable after log_2_ transformation and after adjustment for age, year of hospitalization, and respiratory disease. The findings support the relevance of IL-6 as a marker of an intensified inflammatory response during hospitalization but do not confirm its role as a standalone, validated prognostic tool measured at hospital admission.

The cutoff point of 94.3 pg/mL should be regarded as an exploratory threshold determined for the analyzed cohort rather than as a value recommended for clinical decision-making. Because of the selective nature of IL-6 measurements, the lack of standardized sampling time, and the retrospective nature of the study, the findings require confirmation in independent cohorts.

Further studies should include standardized IL-6 measurement protocols, clearly defined sampling time points, independent validation, and more detailed clinical data, including respiratory parameters, treatment, measures of disease severity, and the course of clinical deterioration.

## Figures and Tables

**Table 1:** Selected characteristics of the patient population in the consecutive years. Categorical variables are presented as counts and percentages, and continuous variables as medians and quartiles.

Variable	Value	
Year of observation	2021	2022
	679 (55.93%)	535 (44.07%)
Sex	women	men
	551 (45.39%)	663 (54.61%)
In-hospital death	yes	no
	230 (18.95%)	984 (81.05%)
History of cancer	yes	no
	94 (7.74%)	1120 (92.26%)
Diabetes	yes	no
	301 (24.79%)	913 (75.21%)
Cardiovascular disease	yes	no
	840 (69.19%)	374 (30.81%)
Respiratory disease	yes	no
	307 (25.29%)	907 (74.71%)
Age [years]	69.0 (59.0–80.0)	
Length of hospital stay [days]	10.0 (7.0–14.0)	
IL-6 [pg/mL]	33.2 (12.3–118.2)	

**Table 2:** Association between the frequency of IL-6 measurement and in-hospital mortality. Variables are presented as counts and percentages; the chi-square test was used.

	IL-6 Level Not Measured	IL-6 Level Measured
death	184 (80.00%)	46 (20.00%)
survival	859 (87.30%)	125 (12.70%)

*p*-value = 0.0042.

**Table 3:** Associations between the frequency of IL-6 measurement and the analyzed patient characteristics. Categorical variables are presented as counts and percentages, and continuous variables are presented as medians and quartiles. The chi-square test was used for categorical variables and the Mann–Whitney test for continuous variables.

Variable	IL-6 Level Not Measured	IL-6 Level Measured	*p*-Value
Year 2021	538 (79.23%)	141 (20.77%)	<0.0001
Year 2022	505 (94.39%)	30 (5.61%)	<0.0001
Women	498 (90.28%)	53 (9.62%)	<0.0001
Men	545 (82.20%)	118 (17.8%)	<0.0001
Positive history of cancer	85 (90.43%)	9 (9.57%)	0.1905
Negative history of cancer	958 (85.54%)	162 (14.46%)	0.1905
Coexisting diabetes	252 (83.72%)	49 (16.28%)	0.2072
No diagnosis of diabetes	791 (86.64%)	122 (13.36%)	0.2072
Coexisting cardiovascular disease	719 (85.60%)	121 (14.40%)	0.6320
No history of cardiovascular disease	324 (86.63%)	50 (13.37%)	0.6320
Coexisting respiratory disease	225 (73.29%)	82 (26.71%)	<0.0001
No history of respiratory disease	818 (90.19%)	89 (9.81%)	<0.0001
Patient age [years]	70.0 (60.0–81.0)	64.0 (51.0–71.0)	<0.0001
Length of hospital stay [days]	10.0 (7.0–14.0)	14.0 (10.0–23.0)	<0.0001

**Table 4:** Association between IL-6 level and in-hospital mortality. Variables are presented as counts and percentages; the chi-square test was used.

Characteristic	IL-6 < 94.3	IL-6 ≥ 94.3
death	21 (45.7%)	25 (54.3%)
survival	99 (79.2%)	26 (20.8%)

*p*-value < 0.0001.

**Table 5:** Associations between IL-6 values and the analyzed patient characteristics. Categorical variables are presented as counts and percentages, and continuous variables are presented as medians and quartiles. The chi-square test was used for categorical variables and the Mann–Whitney test for continuous variables.

Variable	IL-6 < 94.3	IL-6 ≥ 94.3	*p*-Value
Year 2021	98 (69.5%)	43 (30.5%)	0.6772
Year 2022	22 (73.3%)	8 (26.7%)	
Women	41 (77.4%)	12 (22.6%)	0.1688
Men	79 (66.9%)	39 (33.01%)	
Positive history of cancer	5 (55.6%)	4 (44.4%)	0.3246
Negative history of cancer	115 (71.0%)	47 (29.0%)	
Coexisting diabetes	34 (69.4%)	15 (30.6%)	0.8865
No diagnosis of diabetes	86 (70.5%)	36 (29.5%)	
Coexisting cardiovascular disease	79 (65.3%)	42 (34.7%)	0.1192 *
No history of cardiovascular disease	41 (82.0%)	9 (18.0%)	
Coexisting respiratory disease	43 (52.4%)	39 (47.6%)	<0.0001 *
No history of respiratory disease	77 (86.5%)	12 (13.8%)	
Age [years]	61 (48.5–70.0)	68 (60.0–72.0)	0.0064
Length of hospital stay [days]	14 (9.0–21.5)	16 (10.0–28.0)	0.1881

* Result after applying the Bonferroni correction for multiple testing (k = 4 comorbidities).

**Table 6:** Univariable models and the multivariable model assessing the odds of in-hospital death.

Variable	Beta	OR	95% CI	*p*-Value
Univariable models				
Wiek	0.039	1.040	1.013–1.068	0.004
Log_2_ IL-6	0.368	1.445	1.231–1.697	<0.001
Rok 2021 (vs. 2022)	1.373	3.949	1.136–13.722	0.031
Respiratory disease	2.168	8.744	3.752–20.380	<0.001
Multivariable model				
Age	0.056	1.058	1.020–1.097	0.002
Log_2_ IL-6	0.243	1.275	1.075–1.512	0.005
Year 2021 (vs. 2022)	2.123	8.354	2.053–33,991	0.003
Respiratory disease	2.068	7.914	3.012–20,795	<0.001

## Data Availability

All data used in this publication are sourced from the Optmed NXT v.2.11.7 (COMARCH/Mednow) hospital system and are available in Copernicus’s database.
